# Prognosis in autoimmune encephalitis: Database

**DOI:** 10.1016/j.dib.2018.11.020

**Published:** 2018-11-13

**Authors:** James Broadley, Udaya Seneviratne, Paul Beech, Katherine Buzzard, Helmut Butzkueven, Terence O’Brien, Mastura Monif

**Affiliations:** a Department of Neuroscience, Monash University, Melbourne, Australia; b Department of Neuroscience, Monash Health, Melbourne, Australia; c Department of Radiology, Alfred Health, Melbourne, Australia; d Department of Radiology, Monash Health, Melbourne, Australia; e Department of Neurosciences, Eastern Health, Melbourne, Australia; f Department of Neurology, Melbourne Health, Melbourne, Australia; g Department of Neurology, Alfred Health, Melbourne, Australia

## Abstract

Autoimmune encephalitis is a rare and debilitating disease. An important question in clinical neurology is what factors may be correlated with outcomes in autoimmune encephalitis. There is observational data describing statistical analyses on such variables, but there are no review articles that collaborate and interpret this information. This data in brief article represents the data collection for such a review (Broadley et al., 2018).

Herein we summarize clinical information from 44 research articles, in particular pertaining to outcomes and prognostic variables.

**Specifications table**

**Table:** 

Subject area	*Neurology*
More specific subject area	*Autoimmune encephalitis*
Type of data	*Table*
How data was acquired	*Systematic database search*
Data format	*Filtered and analyzed*
Experimental factors	*Confirmed or suspected cases of autoimmune encephalitis with any antibody subtype*
Experimental features	*Search for relevant publications in major databases. Collection of data from included articles.*
Data source location	*Monash University, Wellington Rd, Clayton, VIC 3800, Australia*
Data accessibility	*Data included in article*
Related research article	*J. Broadley, U. Seneviratne, P. Beech, K. Buzzard, H. Butzkueven, T. O’Brien, M. Monif, Prognosticating autoimmune encephalitis: a systematic review, J. Autoimmun., (In Press 2018).*

**Value of the data**

• This data provides a summary of relevant publications in the field, including antibody types, outcome measures, prognosis variables and the quality of this information
• Clinicians seeking to examine research articles for particular prognosis variables or in certain antibody subtypes can review this data to answer these questions easily
• This data provides summaries of demographics, typical symptomatology, rates of tumor association and routine investigation findings for key antibody types
• Clinicians can see rates of good and poor outcomes with respect to antibody subtype and the outcome measure of interest

## 1 Data

The data provided here is a summary of information in research articles describing prognosis in autoimmune encephalitis. This data includes entirely clinical information, with a focus on outcomes and prognosis variables. All causes of autoimmune encephalitis were included in the search, but most of the included articles described cases with antibodies directed to one of the cell surface antigens; NMDAR (*N*-methyl-D-aspartate receptor), VGKC (voltage-gated potassium channel) or GABAb (γ-aminobutyric acid receptor B). Other important abbreviations include MRI (magnetic resonance imaging), CSF (cerebrospinal fluid) and EEG (electroencephalogram). A glossary of terms is provided to aid the interpretation of the following data tables.

Table S1 provides a detailed description of all the research articles that met the inclusion criteria. For each publication we document the type, number of patients, antibody profiles, clinical syndromes, demographics, routine investigation findings, outcome measures, prognosis variables analyzed and the results of this analysis. There is also an objective assessment of quality of each publication, as listed in the final column.

Table 1, Table 2, Table 3, Table 4 summarize clinical information in the research articles divided into each major antibody group. This included cases of anti-NMDAR encephalitis (Table 1), anti-VGKC encephalitis (Table 2) and anti-GABAb encephalitis (Table 2), as well as cases with intracellular antibodies (Table 3). The information listed includes the rates of cardinal symptoms (cognitive impairment, seizure and psychosis), rates of underlying tumor diagnoses, percentage receiving immunotherapy, duration of follow-up and percentage to have good clinical outcomes for each article. Two publications were designed to examine cases without any of the aforementioned antibodies and therefore are only included in Supplementary Table S1 [37], [45].

**Table 1:** Summary of anti-NMDAR encephalitis cases.

**Paper**	**Patients**	**% Seizure**	**% Cognitive change**	**% Psychosis**	**% Tumour**	**%Received IT**	**No followed**	**Median follow-up (months)**	**%Good outcome**
Byun et al. [9]	11	54.5	54.5	72.7	12.5	72.7	11	3	63.6
Byun et al. [10]	17	100	52.9	76.5	0	100	17	6	76.5
Chi et al. [12]	96	80.2	NR	90.6	13.5	95.8	96	24.5	88.5
Constantinescu et al. [13]	4	100	75	75	25	100	4	12	75
Dalmau et al. [15]	100	76	23	77	59.2	92	100	17	75
de Montmollin [16]	77	81.6	NR	NR	47.4	97.4	76	6	56.6
Duan et al. [17]	28	NR	NR	NR	25	96.4	28	6	89.3
Dubey (Journal of Neuroimmunology) [18]	16	75	NR	NR	12.5	Unclear	16	Unclear	62.5
Dubey et al. [19]	7	100	NR	NR	28.6	100	7	Unclear	42.9
Dubey et al. [20]	6	100	NR	NR	NR	Unclear	NA	NA	Unclear
Finke et al. [21]	40	77.5	NR	NR	NR	Unclear	NA	NA	Unclear
Gabilondo et al. [24]	25	NR	NR	NR	20	84	25	20	80
Gresa-Arribas et al. [25]	45	NR	NR	NR	38.8	Unclear	NA	26	Unclear
Harutyunyan et al. [26]	3	NR	NR	NR	NR	Unclear	NA	NA	Unclear
Iizuka et al. [27]	15	93.3	NR	80	33.3	86.7	15	68	86.7
Irani et al. [28]	44	81.8	90.9	77.3	20.5	79.5	44	16	70.4
Jang et al [31]	15	NR	NR	NR	NR	100	15	1	46.7
Lee et al 2016 (Neurology) [33]	27	NR	NR	NR	18.5	100	NA	NA	Unclear
Lee et al 2016 (Neurotherapeutics) [34]	26	NR	NR	NR	23.1	100	NA	NA	Unclear
Leypoldt et al. [35]	167	NR	NR	NR	NR	100^a^	137	8	58.4
Lim et al. [36]	32	50	34.4	68.6	27.3	Incomplete	21	4	63.6
Quek et al. [39]	1	100	0	0	0	100	1	5	100
Titulaer et al. (Lancet Neurology) [42]	577	NR	NR	NR	39.5	92.2^a^	501	6	78.6
Titulaer et al. (Neurology) [43]	31	48.4	100	NR	22.6	Incomplete	29	24	72.4
Wang et al. [46]	43	86	NR	95.3	2.3	83.7	38	4	71.1
Wang et al. [47]	51	84.3	31.4	90.2	7.8	88.2	51	12	80
Zhang et al. [48]	62	74.2	8.1	43.5	8.1	100	62	6	87.1
**Mean/cumulative**	**1566**	**81.27**	**47.02**	**70.56**	**22.07**	**93.13**	**1294**	**13.76**	**72.61**

a Only reported immunotherapy use in the patients that had follow-up.

**Table 2:** Summary of anti-VGKC encephalitis cases.

**Paper**	**Patients**	**% Seizure**	**% Cognitive change**	**% Psychosis**	**% Tumour**	**%received IT**	**No followed**	**Median follow-up (months)**	**%Good outcome**
Arino et al. [5]	76	88.2	100	30.3	6.6	100	48	24	70.8
Aurangzeb et al. [6]	16	100	93.8	NR	NR	Unclear	16	24	81.3
Bataller et al. [7]	5	NR	NR	NR	20	80	5	Unclear	100
Butler et al. [8]	19	73.7	100	31.6	NR	100	17	Unclear	70.6
Byun et al. [10]	17	100	52.9	47.1	0	100	17	6	82.4
Constantinescu et al. [13]	1	100	0	0	0	100	1	12	100
Dubey et al.(Journal of Neuroimmunology) [18]	9	88.9	NR	NR	44.4	Unclear	9	Unclear	44.4
Dubey et al. (Seizure) [19]	8	100	NR	NR	37.5	100	8	Unclear	62.5
Dubey et al. [20]	18	100	NR	NR	NR	Unclear	NA	NA	Unclear
Finke et al. [22]	30	93.3	100	NR	10	96.7	30	23.3	80
Flanagan et al. [23]	11	54.5	100	36.4	18.2	100	11	22	90.9
Harutyunyan et al. [26]	6	NR	NR	NR	NR	Unclear	NA	NA	Unclear
Irani et al. [29]	26	34.5	NR	NR	41.4	Unclear	26	unclear	65.4
Irani et al. [30]	10	100	80	NR	10	100	10	18	100
Jang et al. [31]	15	NR	NR	NR	NR	100	NA	NA	Unclear
Lee et al. (Neurology) [33]	3	NR	NR	NR	0	100	NA	NA	Unclear
Lee et al. (Neurotherapeutics) [34]	3	NR	NR	NR	0	100	NA	NA	Unclear
Malter et al. [38]	10	100	90	NR	0	100	9	17	100
Quek et al. [39]	18	100	61.1	16.7	16.7	88.9	18	Unclear	100
Shin et al. [40]	14	100	85.7	0	7.1	100	12	4.5	91.7
Thompson et al. [41]	103	100	78.6	12.6	7.8	95.1	NA	NA	Unclear
Toledano et al. [44]	12	100	NR	NR	NR	100	12	22.5	100
**Mean/cumulative**	**430**	**90.18**	**78.51**	**21.84**	**13.73**	**97.69**	**249**	**17.33**	**83.75**

**Table 3:** Summary of anti-GABAb encephalitis cases.

**Paper**	**Patients**	**% Seizure**	**% Cognitive change**	**% Psychosis**	**% Tumour**	**% Received IT**	**No Followed**	**Median follow-up (months)**	**% Good outcome**
Byun et al. [10]	3	100	NR	NR	66.7	100	3	6	66.7
Chen et al. [11]	11	100	90.9	27.3	27.3	100	11	8.6	63.6
Constantinescu et al. [13]	1	100	0	100	0	100	1	12	100
Dubey et al. (Journal of Neuroimmunology) [18]	3	66.6	NR	NR	33.3	Unclear	3	Unclear	66.7
Dubey et al. (Seizure) [19]	2	100	NR	NR	50	100	2	Unclear	50
Harutyunyan et al. [26]	2	NR	NR	NR	NR	Unclear	NA	NA	Unclear
Jang et al. [31]	1	NR	NR	NR	NR	100	NA	NA	Unclear
Lancaster et al. [32]	15	100	100	26.7	46.7	73.3	14	6	57.1
**Mean/cumulative**	**38**	**94.43**	**63.63**	**51.33**	**37.33**	**95.55**	**34**	**8.15**	**67.35**

**Table 4:** Summary of encephalitis cases with intracellular antibodies.

**Paper**	**Patients**	**Antibody types**	**% Seizure**	**% Cognitive change**	**% Psychosis**	**% Tumour**	**%Received IT**	**No followed**	**Median follow-up (months)**	**%Good outcome**
Bataller et al. [7]	14	7 Hu; 6 Ma2; 1 atypical	NR	NR	NR	100	100	12	Unclear	25
Byun et al. [10]	4	2 Ma2 (also had Ta), 1 Yo; 1 amphiphysin	100	NR	NR	50	100	4	6	75
Constantinescu et al. [13]	2	2 Ma2	100	50	50	0	100	2	12	100
Dalmau et al. [14]	38	38 Ma2 (15 also had Ma1)	31.6	68.4	NR	89.5	50	33	30	33.3
Dubey et al. (Journal of Neuroimmunology) [18]	1	1 Hu	0	NR	NR	0	unclear	1	Unclear	0
Dubey et al. [20]	4	2 Hu; 1 CV2; 1 Ri	100	NR	NR	NR	Unclear	NA	NA	Unclear
Flanagan et al. [23]	2	1 Hu; 1 amphiphysin	50	100	50	100	100	2	34	50
Harutyunyan et al. [26]	4	1 Hu; 1 Yo; 1 Ma2 (also Ma1); 1 CV2	NR	NR	NR	NR	Unclear	NA	NA	Unclear
Lee et al. (Neurology) [33]	15	4 Ma2 (also Ta); 2 Yo; 2 Hu; 7 amphiphysin	40	NR	NR	20	100	15	Unclear	60
Lee et al. (Neurotherapeutics) [34]	2	2 amphiphysin	NR	NR	NR	0	100	NA	NA	Unclear
Quek et al. [39]	3	2 CV2; 1 Ma2 (also had Ma1)	100	66.7	0	33.3	100	3	Unclear	66.7
Toledano et al. [44]	1	1 Ma2 (also had Ma1)	100	NR	NR	NR	100	1	32	0
**Mean/cumulative**	**90**		**69.07**	**71.28**	**33.33**	**43.64**	**94.44**	**73**	**22.8**	**45.56**

## 2 Experimental design, materials and methods

Relevant publications were identified by searching abstracts in MEDLINE, Embase, PsychInfo and PubMed databases from their inception to 30/04/2018. Search terms including autoimmune encephalitis, autoimmune antibody subtypes, outcome and prognosis were combined with Boolean operators (see Table 1 in Ref [1]). Furthermore, the reference lists of included publications were also examined to identify additional articles undetected in the initial search.

Research articles were eligible for this review if they were original research on patients diagnosed with autoimmune encephalitis that provided a statistical analysis of factors that correlated with the patient outcome. Publications were included based on cases with features of encephalitis that were suspected or confirmed to have an autoimmune cause. Publication focusing on other antibody associated CNS or non-CNS syndromes, such as paraneoplastic cerebellar degeneration, stiff person syndrome, isolated myelitis or paraneoplastic neuropathy were excluded. In addition, articles that reported such cases in a wider cohort of patients with encephalomyelitis, where the statistics could not be isolated for cases of encephalitis only, were also excluded. Inclusion criteria were English language publications and the availability of full text. Animal publication, grey literature and case series reporting less than 10 patients were excluded. Data performed solely in children were also excluded. Finally, articles where autoimmune encephalitis was a subset of a larger publication on encephalitis due to multiple aetiologies were excluded.

Each publication underwent a detailed review, during which the following details were extracted: number of patients, antibody subset, clinical syndrome, age, sex, abnormal investigation findings, outcome measures, factors tested for outcome correlation and publication results (Table S1). Papers that tested early magnetic resonance imaging (MRI) findings and/or cerebrospinal fluid (CSF) characteristics as possible markers for prognosis were noted. The CSF parameters considered applicable were those identified by routine testing, such as protein, glucose, white cell count, or differential cell counts. The MRI abnormalities recorded were those likely due to encephalitis, or any MRI abnormality if these details were not specified. Articles listed as having "Multiple" antibodies include both seropositive and seronegative cases unless stated otherwise. All ages are reported in years. Average age is given as what was reported in paper (mean, median or mode). Ranges were also as reported in paper and given to nearest whole number (actual range, upper/lower quartiles or standard deviation). Where both were included in the data, only the actual range was recorded. Abnormal CSF is reported based on the presence of either elevated protein or leukocytosis on initial CSF analysis. Abnormal MRI is reported based on the presence of one or more of T2/FLAIR changes, contrast enhancement, or leptomeningeal enhancement on initial MRI. Abnormal EEG is reported based on the presence of epileptiform discharges, inter-ictal spikes, focal slowing or extreme delta brush pattern on EEG in initial admission. Numbers in brackets in the EEG column are inclusive of patients with any electrographic abnormality (including generalised slowing). In articles that did not include this level of detail, we took their definition of abnormal CSF/MRI/EEG. Percentages are taken in relation to the reported numbers of participants to have had each test. In circumstances where both values (eg elevated CSF protein and white cell count) were reported but not the number of participants that had one or the other, we took the highest single value. Relationships are based on highest statistical test performed; multivariate regression analysis if available, univariate analysis if not. Therefore, significant relationship on univariate analysis, subsequently disproven on multivariate analysis is not listed here as a correlation.

For the most common antibody subtypes details were obtained on seizure frequency, cognitive impairment, psychosis, underlying neoplasia, immunotherapy, follow-up timeframes and reported outcomes (Table 1, Table 2, Table 3, Table 4). Reported rates of immunotherapy usage were in the original cohort unless otherwise stated. In papers where there was loss to follow-up, this data describes outcomes in the most complete group of patients, even if that follow-up was significantly shorter than the longest follow-up. This was meant to reduce the error in the collection and reporting of data. This data used the outcomes that were defined as good or favorable by the authors of each research article, or where patients were left with no or only mild deficits when no definition was given. For articles with an adequate description of relevant outcome data, this data includes the patients’ median follow-up timeframe, the numbers of patients with follow-up information and the proportion to have a positive outcome. Data was excluded if any information was unclear or incomplete, in particular noting if it was unclear for the antibody subset of interest. A breakdown of constituent antibodies was documented in articles that included intracellular autoantibodies. Using the information obtained from the articles, this dataset includes the calculated average rates of seizure presentations, cognitive impairment, psychosis, tumor diagnoses and immunotherapy usage as well as the average rates of favorable outcomes for the major antibody groups. This information describes seizure of any type, including faciobracial dystonic seizures. Symptoms listed were based on initial symptoms only, not symptoms at relapse. Notably some publications report cognition or psychiatric features together; this data was excluded. Percentage tumor reflects the proportion of diagnosis from patients that were imaged for tumor. The last row in each table represents mean values, except in columns of patient numbers which are cumulative.

Included articles were reviewed independently by two authors (JB & US), and classified based on the publication design. Where discrepancy was found, consensus agreement was reached. The articles were objectively assessed for quality, using the Newcastle-Ottawa Scale for cohort and case-control studies[2], an adapted version of the Newcastle-Ottawa Scale for cross-sectional studies[3], and the quality assessment tool for case series proposed by Moga et al[4]. Each research article was classified as having good, fair or poor quality based on the scoring of these quality assessment tools (see Table 2 in Ref [1]).

## Glossary of terms

AED: anti-epileptic drug,
AMPA: α-amino-3-hydroxy-5-methyl-4-isoxazolepropionic acid,
CASPR2: contractin-associated protein-like 2,
CPS: cognitive performance score,
CRP: C-reactive protein,
CS: corticosteroid,
CSF: cerebrospinal fluid,
CT: computed tomography,
EEG: electroencephalogram,
FBDS: faciobrachial dystonic seizure,
GABAb: γ-aminobutyric acid receptor B,
GAD: glutammic acid decarboxylase,
GCS: Glasgow coma scale,
GFR: glomerular filtration rate,
ICU: intensive care unit,
IgG: immunoglobulin type-G,
IVIg: intravenous immunoglobulin,
IT: immunotherapy,
LFT: liver function test,
LGI1: leucine-rich glioma inactivated protein 1,
MMSE: mini-mental state examination,
MOCA: Montreal Cognitive Assessment,
MRI: magnetic resonance imaging of the brain,
MRS: modified Rankin Score,
MTL: mesial temporal lobe,
NA: not applicable,
NMDA: N-methyl-D-aspartate,
NR: not reported,
PET: positron emission topography,
TPO: thyroid peroxidase,
VGKC: voltage-gated potassium channel,
WBC: white blood cell
