# Supplementary material for: Prognosis in autoimmune encephalitis: Database
Source: Data Brief. 2018 Nov 13;21:2694–703. doi: 10.1016/j.dib.2018.11.020 (PMC6290378; doi:10.1016/j.dib.2018.11.020)
Supplement: Supplementary file 2 — Supplementary material [file mmc2.docx]

**Table S1 Main data extraction table**

| **Study** | **Study type** | **Number of cases** | **Antibody** | **Syndrome** | **Comment** | **Average age (range)** | **%female** | **% abnormal CSF/MRI/EEG** | **Outcome measure/s** | **Variables tested** | **Associated with outcome (poor unless stated otherwise)** | **Paper quality score** |
| --- | --- | --- | --- | --- | --- | --- | --- | --- | --- | --- | --- | --- |
| Arino et al 2016 [5] | Cohort study | 76 | anti-VGKC | Cognitive impairment | anti-LGI1 antibodies; Described 3 syndromes - limbic encephalitis, non-limbic encephalitis, encephalopathy | 61 (32-80) | 34.2 | 22.6/75/NR | CPS (designed by study authors: good ≤1, poor >1); relapse | age, gender, subacute onset (≤3 months, clinical syndrome (encephalopathy vs encephalitis), CPS nadir, hyponatremia, median LGI1 titer (serum and CSF), IgG class in serum at onset, dleay to IT, improvement with 1st line IT, type of IT (CS + IVIg, vs CS or IVIg), second-line IT, long-term IT, relapses | Failure of first line treatment; clinical relapses | 6/9  (Fair) |
| Aurangzeb et al 2017 [6] | Case series | 16 | anti-VGKC | Autoimmune encephalitis | anti-LGI1 antibodies | 67 (53-92) | 18.6 | NR/NR/50 | MRS improvement by ≥1 | cumulative seizure frequency; age; gender; time to "medication" | cumulative seizure frequency | 9/18  (Poor) |
| Bataller et al 2007 [7] | Case series | 39 | Multiple | Autoimmune encephalitis | comparative study between cases with cell surface and intracellular antibodies | (3-67)* | 53.8 | NR/92.3/NR | Functional outcome (complete improvement, partial improvement, stable, dead) | intracellular vs cell surface antibodies | intracelllular antibodies | 10/18  (Poor) |
| Butler et al 2014 [8] | Case series | 19 | anti-VGKC | Limbic encephalitis |  | 60.1 (46-75) | 36.8 | NR/54.5/NR | cognitive tests | serum antibody titer; (other factors tested but since non-significant they were discarded by authors) | serum antibody titer | 12/18  (Poor) |
| Byun et al 2015 [9] | Cohort study | 11 | anti-NMDAR | Autoimmune encephalitis | Controls included following cases: 15 non-specific neurological symptoms and 9 HSV encephalitis | 38 (32-54) | 54.5 | 45.4/36.4/27.3 | MRS good 0-1; poor ≥2 | Variables denoting heart rate variability | Heart rate variability | 7/9  (Good) |
| Byun et al 2016 [10] | Case series | 41 | Multiple | Autoimmune encephalitis | All patients had new onset seizures; all patients seropositive | 43 (18-74) | 48.7 | 50/43.9/41.5 | seizure outcome (remission vs ongoing Sz) | gender, age, seizure duration, FBDS, status epilepticus, abnormal MRI, epileptiform changes on EEG, CSF leukocytosis, elevated CSF protein, MRS <3 on admission, follow-up MRS <3, NMDA receptor antibody, VGKC antibody, GABAb antibody, intracellular antibody, tumor, number of AEDs before IT, maximum number of AEDs, escalation of AED, CS use only, IVIg use only, CS and IVIg combined IT | abnormal MRI | 14/18  (Good) |
| Chen et al 2017 [11] | Case series | 11 | anti-GABAb | Autoimmune encephalitis |  | 51 (18-76) | 27.2 | 72.7/18.2/36.4 (63.6) | MRS (good 0-2, poor >2) | age; presence of status or refractory seizures; presence of small cell lung cancer | age | 11/18  (Poor) |
| Chi et al 2017 [12] | Case series | 96 | anti-NMDAR | Autoimmune encephalitis |  | 24.5 (9-71) | 62.5 | 57.3/44.9^/74.4 | mortality | gender, age, highest temperature, seizure, psychiatric symptoms, latency between symptoms and admission, GCS ≤8, high intracranial pressure, status epilepticus, refractory status epilepticus, tumor, number of complications, multi-organ dysfunction, abnromal EEG, abnormal MRI, hypokalemia, hyponatremia, CSF pleocytosis, leukocytosis, hypoalbuminemia, mechanical ventilation, admission to IT, admission to diagnosis, use of methylprednisolone, use of IVIg, use of second line immunotherapy, no use of immunotherapy, ICU admission, length of hospital stay, complications (pneumonia, respiratory failure, urinary tract infection, circulatory system complications, digestive system complications) | GCS≤8 at admission, ICU admission, number of complications | 13/18  (Poor) |
| Constantinescu et al 2016 [13] | Case series | 25 | Multiple | Autoimmune encephalitis |  | 42 (18-75) | 68 | 80/60/NR | MRS (good 0-2, poor 3-6) | neurofilament light chain protein at all time points; T-tau at all time points; glial fibrillary acidic protein at all time points "There were otherwise no associations between MRI abnormalities at LP1 and LP3 and CSF brain marker levels, CSF immunopathy and CSF inflammatory parameters, the presence of SE at LP1, and outcomes." | neurofilament light chain protein level at all time points; T-tau at LP2 | 12/18  (Poor) |
| Dalmau et al 2004 [14] | Case series | 38 | Anti-Ma2 | Autoimmune encephalitis | Some patients had co-existing anti-Ma1 antibodies; | 34 (22-70) | 31.6 | 71.9/65.7/100 | decrease MRS ≥1 | gender; age >45; unifocal/multifocal CNS involvement; antibody profile (Ma2 vs Ma1 and Ma2); testicular tumor vs other tumor; complete vs incomplete response to tumor therapy; immunotherapy | age>45; multifocal CNS involvement; Ma1 and 2 antibodies; non-testicular tumor; incomplete response to tumor treatment | 13/18  (Poor) |
| Dalmau et al 2008 [15] | Case series | 100 | anti-NMDAR | Autoimmune encephalitis |  | 23 (5-76) | 91 | 95/55/22.8 (77.2) | MMSE and MRS divided into different categories (full recovery = MRS 0/MMSE 29-30, mild deficits = MRS 1-2/MMSE 25-28, severe deficits = MRS >2/MMSE <25); relapses | Early vs late tumor treatment; early tumor treatment vs no tumor | Early tumor treatment associated with reduced relapse rate and better outcome | 11/18  (Poor) |
| de Montmollin et al 2017 [16] | Case series | 77 | anti-NMDAR | Autoimmune encephalitis | All patients had ICU admission | 24 (20-31) | 89.6 | NR/25.3/22.7 (88) | MRS (good 0-2, complete recovery = MRS 0) | age, gender, reason for ICU admission (coma vs seizures vs agitation/confusion vs respiratory failure vs autonomic dysfunction), Knaus Scale A, MRS before symptom onset (0 vs 1), SAPS II score, SOFA score, non-neurologic SOFA score, Glasgow score, interval between symptoms and ICU admission, diagnosis before ICU admission, seizure characteristics (generalised tonic-clonic vs partial complex vs mixed vs other), status epilepticus during ICU stay, refractory status epilepticus during ICU stay, CSF WBC count, CSF protein level, abnormal CT brain, abnormal MRI, EEG characteristics (normal vs epileptic activity vs slowing vs other), tumor imaging (CT vs MRI vs both), delay between ICU admission and tumor imaging, presence of tumor, delay between ICU admission and tumor resection, histological diagnosis (mature ovarian teratoma vs immature ovarian teratoma vs small cell lung cancer vs unrelated to anti-NMDA receptor encephalitis), use of immunotherapy, use of CS, use of IVIg, use of plasma exchange, delay between ICU admission and treatment (1st line treatment, CS, IVIg, plasma exchange), delay between ICU admission and second line treatment, use of second line IT (cyclophosphamide vs rituximab vs cyclophosphamide and rituximab), | **GOOD OUTCOME:** CSF WBC count <50 cells/mm3, early combined immunotherapy with CS and IVIg | 13/18  (Poor) |
| Duan et al 2016 [17] | Case series | 28 | anti-NMDAR | Autoimmune encephalitis | Focus on movement disorders (all patients had some form of movement disorder) | 15 (0.67-38) | 71.4 | NR/NR/NR | MRS (continuous); duration of movement disorders | age; tumor | tumor duration of catatonia and choreoathetosis significantly shorter in patients <10 years | 11/18  (Poor) |
| Dubey et al 2015 (Journal of Neuroimmunology) [18] | Case series | 64 | Multiple | Autoimmune encephalitis |  | 40.4 (2-86) | 43.7 | 63.3/59.4/NR | **improvement in major symptom:** >50% seizure reduction, complete/near complete resolution of memory impairment (MMSE/MOCA >25), complete/near complete resolution of movement disorder, complete/near complete resolution of behavioral changes | delay in diagnosis; delay in IT; time from symptom onset to first clinic appointment; presence of tumor; absence of prodrome; type of IT given (CS vs plasma exchange); hospital where patient was treated | delay in diagnosis; delay in IT; time from symptom onset to first clinic appointment; presence of tumor; absence of prodrome | 14/18  (Good) |
| Dubey et al 2015 (Seizure) [19] | Case series | 34 | Multiple | Autoimmune epilepsy |  | 45# | 35.3 | NR/70.6/100 | 50% reduction seizures | gender; race; age; country vs university hospital; presenting symptom; type of antibody; presence of tumor; type of antibody (cell surface vs GAD or TPO); delay in diagnosis; delay in IT; delay in MRI; delay in EEG; delay in lumbar puncture; pre-treatment seizure frequency; number of AEDs; CSF lymphocytic pleocytosis; EEG ictal and interictal patterns; pattern of MRI changes | tumor; delay in diagnosis; delay in IT; delay in MRI; delay in EEG; delay in lumbar puncture | 13/18  (Poor) |
| Dubey et al 2017 [20] | Case series | 387 | Multiple | Autoimmune epilepsy | Took patients with epilepsy where antibody panel sent (ie. Possible autoimmune epilepsy) | (0.5-87)* | 56.1 | 11.9/18.6/NR | >50% reduction seizures | age, gender, any neural antibody present, cell surface antibody present, intracellular antibody present, median APE score, APE ≥4, median RITE score, RITE ≥7, new onset seizures, neuropsychiatric symptoms, autonomic dysfunction, viral profrome, FBDS present, refractory seizures prior to IT, CSF protein >50mg/dl, CSF cell count >5 cells/dl, CSF oligoclonal bands >4, MTL FLAIR/T2 changes, MTL sclerosis, presence of tumor, time to IT, symptom onset to IT <6 months, use of methylprednisolone, use of IVIg, use of plasma exchange, use of second line IT | **GOOD OUTCOME:** male; any neural Ab present; cell surface antibody present; APE score ≥4; RITE score ≥7; new onset seizures; neuropsychiatric changes; autonomic instability; presence of FBDS; shorter delay in IT; interval of symptom onset to IT <6 months; IV methylprednisolone use; second line agent use | 14/18  (Good) |
| Finke et al 2016 [21] | Cohort study | 40 | anti-NMDAR | Autoimmune encephalitis | 25 healthy controls | 28 (18-67) | 90 | NR/NR/NR | MRS (continuous); cognitive tests | follow-up duration; serum/csf antibody titers | longer follow-up associated with better MRS, but not cognitive outcome | 9/9  (Good) |
| Finke et al 2017 [22] | Cross-sectional study | 30 | anti-VGKC | Autoimmune encephalitis | anti-LGI1 antibodies; 27 age matched controls | 65.7 (53-78) | 36.7 | NR/73.3/25.9 (70.4) | MRS (continuous); cognitive tests | delay in IT, use of second line IT, antibody titres, time to follow-up | delay in immunotherapy associated with poor cognitive outcome; use of second IT associated with poor cognitive and MRS outcome | 9/10  (Good) |
| Flanagan et al 2010 [23] | Case series | 72 | Multiple | Autoimmune dementia |  | 58 (46-70) | 58.3 | 28.6/22.2/18.2 | short test of of mental status; multiple cognitive tests | headache; VGKC antibodies alone; TPO antibody, family history of dementia, high Kokmen score before treatment; TPO antibody; delayed treatment subacute onset; fluctuating course; tremor; shorter delay to treatment; cation Ab pos; abnormal CSF; any neural antibody | **POOR OUTCOME:** family history of dementia, high Kokmen score before treatment; TPO antibody; delayed treatment **GOOD OUTCOME:** subacute onset; fluctuating course; tremor; shorter delay to treatment; cation Ab pos; abnormal CSF; any neural antibody | 11/18  (Poor) |
| Gabilondo et al 2011 [24] | Case series | 25 | anti-NMDAR | Autoimmune encephalitis |  | 23 (0.7-53) | 88 | 80/60/90.9 | clinical relapse | age, gender, presence of ovarian teratoma, clinical syndrome (typical encephalitis vs epilepsy), CSF WWC >5cells/mm3, abnormal MRI, abnormal EEG, No use of IT, type of IT (CS vs CS and IVIg), plasma exchange, rituximab, cyclophosphamide, tumor removal (where appropriate), duration of follow-up | No IT at first episode | 12/18  (Poor) |
| Gresa-Arribas et al 2014 [25] | Cohort study | 45 | anti-NMDAR | Autoimmune encephalitis | 250 cases and 100 encephalopathic controls compared for sensitivity and specificity of antibody testing | NR | NR | NR/NR/NR | MRS (good 0-2, poor 3-6) | NMDA titres (serum and/or CSF) at 3 different time points. | **Monophasic illness only:** Serum and CSF titres higher in patients with poor outcomes | 3/9  (Poor) |
| Harutyunyan et al 2017 [26] | Case series | 32 | Multiple | Autoimmune encephalitis |  | 64 (54-73) | 31.3 | 61.3/70/86.7 | MRS (good 0-3, poor >3) | age, gender, comorbidities (hypertension, malignancy, hyperlipidemia, nicotine abuse, autoimmune disease, type 2 diabetes mellitus, hypothyroidism, alcohol abuse), Charlson's comorbidity illness, latency between symptoms and hospital admission, presenting symptoms (altered GCS, seizures, memory loss, movement disorder, headache, speech impairment), length of hospital stay, any neural antibody, ICU admission, latency between symptoms and diagnosis, anaemia, serum hypoproteinemia, serum leukocytosis, elevated serum gamma-GT, abnormal MRI, abnormal EEG, inflammatory CSF, red blood cells in CSF, CSF pleocytosis, elevated CSF IgG, CSF oligoclonal bands, elevated vitamin B12, elevated folic acid levels, latency between symptoms and IT, latency between admission and IT, type of IT (CS, IVIg, plasma exchange, rituximab, cyclophosphamide), improvement after 1st line IT, improvement after 2nd line IT | anaemia | 11/18  (Poor) |
| Iizuka et al 2016 [27] | Case series | 15 | anti-NMDAR | Autoimmune encephalitis |  | 21 (14-46) | 66.7 | 93.3/53.3/44.4 (55.5) | MRS (good 0-2) | cerebellar atrophy; diffuse cerebral atrophy without cerebellar atrophy; ventilator support; serious complications; prolonged hospitalisation | cerebellar atrophy | 12/18  (Poor) |
| Irani et al 2010 [28] | Case series | 44 | anti-NMDAR | Autoimmune encephalitis |  | 22 (2-49) | 70.5 | 68.1/11.7/50(79.5) | MRS (continuous) | Demographic and tumor (adult with tumor vs adult without tumor vs child); use of IT; Delay in IT; delay in cancer treatment; type of IT (CS alone vs combined with another treatment) | no IT treatment; time to IT (non-paraneoplastic cases); time to cancer treatment (paraneoplastic cases) | 10/18  (Poor) |
| Irani et al 2012 [29] | Cohort study | 29 | anti-VGKC | Morvan syndrome | cases mostly have anti-LGI1 antibodies, anti-CASPR2 antibodies or both; 3 patients seronegative | 57 (19-80) | 6.9 | 47.6/8/11.8 (64.7) | mortality | tumor; clinical syndrome (Morvan vs limbic encephalitis or neuromyotonia) | tumor, Morvan syndrome | 4/9  (Poor) |
| Irani et al 2013 [30] | Case series | 10 | anti-VGKC | FBDS |  | 68 (28-92) | 50 | 0/25/40 | cognitive impairment, time to MRS 1 | administration of IT, time to IT, time to AED | Administration of IT prevented onset of cognitive impairment Delay in IT resulted in longer time to MRS 1 | 14/18  (Good) |
| Jang et al 2018 [31] | Case series | 32 | Multiple | Autoimmune encephalitis | All patients seropositive | 47.1 (26-68) | 50 | 55.6/40.6/34.4 | MRS improvement by ≥1 at 1 month; MRS long term (good 0-2) | **Short term improvement:** age, gender, NMDA receptor antibody, LGI1 receptor antibody, other antibodies (GABAb, CASPR2, AMPA2), tumor, BMI (low, normal, high), initial severity ≥4, low serum albumin (<4.0g/dL), total cholesterol, LFT abnormality, C-reactive protein elevation, creatine kinase, GFR, CSF WBC >5, elevated CSF protein, abnormal EEG, abnormal MRI, time to IT, type of IT (IVIg vs CS and IVIg), use of second line IT, latency between 1st and 2nd line IT **Long term outcome:** low serum albumin (<4.0g/dL) | low albumin associated with poorer initial response; low albumin associated with slower MRS recovery (but achieve same MRS); | 12/18  (Poor) |
| Lancaster et al 2010 [32] | Cohort study | 15 | anti-GABAb | Limbic encephalitis |  | 62 (24-75) | 46.7 | 9073.3/75 (91.7) | Neurological improvement, defined as ability to function independently or with little assistance when they returned home (equivalent to MRS ≤3) | IT and/or complete tumour treatment | no IT and/or incomplete tumour treatment | 6/9  (Poor) |
| Lee et al 2016 (Neurology) [33] | Cohort study | 80 | Multiple | Limbic encephalitis | 81 non-rituximab treated patients used as control | 43.1 (16-80) | 47.5 | NR/64/36.6 (67.1) | MRS (good 0-2); MRS improvement | gender, age, time to IT, IT given in ≤1 month, number of cycles of 1st line IT, ≥2 cycles 1st line IT, time to rituximab, CSF lymphocyte count, CSF protein level, presence of any antibody, cell surface antibody, intracellular antibody, tumor, symptoms (memory/exective function impairment, language dysfunction, psychiatric symptoms, cerebellar dysfunction, movement disorder, autonomic dysfunction, seizure, altered conscious state), favorable MRS at 1st line IT, MRS nadir ≥4, ICU admission, favorable MRS at rituximab commencement, relapse after 1st line IT, use of IVIg, use of CS, use of plasma exchange, repetitive use of same IT, combination 1st line IT, simultaneous combination first line IT, abnormal MRI, presence of T2 lesions on MRI, T2 lesions in MTL on MRI, EEG slowing, EEG spikes, early rituximab treatment, rituximab with 1.5 months from onset, rituximab >14 months from onset, concomitant immune modulation | MRS improvement: response to first line IT, additional monthly rituximab Unfavourable MRS: MRS nadir ≥4, response to first line IT, additional monthly rituximab | 9/9  (Good) |
| Lee et al 2016 (Neurotherapeutics) [34] | Cohort study | 91 | Multiple | Autoimmune encephalitis | patients had already failed rituximab | 38.8 (5-79) | 52.7 | NR/60.4/48.4 | MRS improvement ≥2 points, favorable MRS (good ≤2) | gender, age, presence of antibody, NMDA receptor antibodies, symptoms (seizures, memory impairment, language dysfunction, behavioural/psychiatric symptoms, movement disorder, autonomic dysfunction, altered conscious state, cerebellar dysfunction, central hypoventilation), CSF lymphocytosis, elevated CSF protein, white matter T2 lesions on MRI, T2 lesions in MTL on MRI, EEG epileptic discharges, early tocilizumab treatment, MRS≥4 at tocilizumab commencement | No association found | 9/9  (Good) |
| Leypoldt et al 2015 [35] | Cohort study | 167 | anti-NMDAR | Autoimmune encephalitis | control cases included: 25 non-inflammatory negative controls/9 neuroborreliosis positive controls | NR | NR | NR/NR/NR | MRS (good 0-2, poor>2) | CSF CXCL13 at onset and between 2-6 months after immunotherapy onset | CXCL13 at onset positively correlated poor outcome; CXCL13 at 2-6 months stronger correlation with poor outcome; CXCL13 obtained at remission positively correlated with relapse | 5/9  (Poor) |
| Lim et al 2014 [36] | Case series | 32 | anti-NMDAR | Autoimmune encephalitis |  | 41.5 (19-80) | 37.5 | 69.2/51.6/41.4 (75.9) | MRS good 0-2, poor 3-6 | loss of consciousness; autonomic dysfunction; central hypoventilation; delay in treatment; age; symptoms; tumor; MRS nadir before immunotherapy | loss of consciousness; autonomic dysfunction | 8/18  (Poor) |
| Litmeier et al 2016 [37] | Case series | 11 | anti-TPO | SREAT | Majority had co-existing thyroid disease; two patients ultimately received alternate diagnoses | 53.8 (22-82) | 84.6 | 83.3/45.5/NR | Glasgow Outcome Scale; | age; TPO titer | age; low TPO titer | 10/18  (Poor) |
| Malter et al 2010 [38] | Case series | 19 | anti-GAD/anti-VGKC | Limbic encephalitis | Comparative study between anti-GAD and anti-VGKC encephalitis | (17-73)* | 57.9 | 26.3/100/NR | seizure freedom; number of AEDs; cognitive testing | GAD vs VGKC | cases anti-VGKC encephalitis had more seizure remission and less AED use | 12/18  (Poor) |
| Quek et al 2012 [39] | Case series | 32 | Multiple | Autoimmune epilepsy |  | 56 (5-79) | 59.4 | 56.7/53.1/84.3 (90.6) | seizure outcome (no change, improvement, freedom) | delay in IT | delay in immunotherapy | 10/18  (Poor) |
| Shin et al 2013 [40] | Case series | 14 | anti-VGKC | Autoimmune encephalitis | anti-LGI1 antibodies | 60.5 (41-78) | 42.9 | 23.1/71.4/61.5 (76/9) | MRS (continuous); MRS (good = 0); relapse | treatment regimen (CS vs CS and IVIg); delay in IT; MTL hypermetabolism on PET; unilateral vs bilateral MTL hypermetabolism on PET; basal ganglia hypermetabolism on PET, MTL lesion on MRI | **MRS (continuous):** CS only treatment, delay in IT; MTL hypermetabolism on PET; bilateral hypermetabolism **unfavorable MRS:** CS only treatment, MTL hypermetabolism on PET, bilateral hypermetabolism **relapse:** no association found | 11/18  (Poor) |
| Thompson et al 2018 [41] | Case series | 103 | anti-VGKC | FBDS | anti-LGI1 antibodies | 64 (22-92) | 37.9 | 17.6/55.3/51.5 | MRS (continuous) | age; gender; FBDS frequency; time to AED; time to IT; cognitive impairment | time to IT; cognitive impairment | 14/18  (Good) |
| Titulaer et al 2013 (Lancet Neurology) [42] | Cohort study | 577 | anti-NMDAR | NR | No other clinical information used in selection criteria other than antibody positivity (serum or CSF) | 21 (0.67-85) | 80.9 | 78.6/33.3/89.6 | MRS (good 0-2, poor 3-5); first line treatment failure; relapse | MRS: MRS nadir, ICU admission, follow-up duration, delay in treatment initiation, tumor, age, gender 1st line IT failure: ICU admission, delay in treatment, follow-up duration, second line treatment Relapse: use of IT, presence of tumor | MRS: ICU admission, delay in treatment, MRS nadir 1st line IT failure: ICU admission, delay in treatment relapse: use of IT, tumor (had a lower rate of relapse) | 8/9  (Good) |
| Titulaer et al 2013 (Neurology) [43] | Cohort study | 31 | anti-NMDAR | Autoimmune encephalitis | late onset (age≥45) | 52 (45-84) | 54.8 | 79/53/86 | MRS (good 0-2) | age group; delay in treatment; ICU admission; follow-up | older age group; delay in treatment; ICU admission; shorter follow-up (latter may be affected by mortality) | 5/9  (Fair) |
| Toledano et al 2014 [44] | Case series | 29 | Multiple | Autoimmune epilepsy |  | 53 (2-79) | 55.2 | 65.5/62.1/86.2 | seizure freedom/improvement | gender, age, duration of seizures, seizure types (focal with awareness, focal without awareness, generalised tonic-clonic, FBDS, multiple types), number of AEDs, changes to AEDs, seizure frequency, subacute presentation, coexisting autoimmune disease, family history of autoimmune disease, past history of neoplasm, viral prodrome, inflammatory MRS changes, elevated CSF protein (>35mg/dL), elevated CSF WBC count, CSF oligoclonal bands or elevanted IgG, presence of any neuronal antibody, cell surface antibody, GAD65 antibody, intracellular antibody | Delay in IT; non-cell surface antibody; single seizure type | 11/18  (Poor) |
| von Rhein et al 2017 [45] | Case series | 28 | Antibody negative | Limbic encephalitis | Suspected Ab-negative autoimmune limbic encephalitis | 45 (29-61) | 28.5 | 17.6/100/NR | cognitive testing; Beck Depression Inventory | Gender; IT discontinuation; seizure control; | **Cognitive outcome:** male **Depression outcome:** discontinuation of IT | 11/18  (Poor) |
| Wang et al 2015 [46] | Case series | 43 | anti-NMDAR | Autoimmune encephalitis |  | 23 (9-39) | 55.8 | 58.1/NR/NR | MRS (good 0-2) | serum positivity; CSF pressure abnormality; CSF cell count abnormality; CSF protein abnormality | No association found | 11/18  (Poor) |
| Wang et al 2016 [47] | Case series | 51 | anti-NMDAR | Autoimmune encephalitis |  | 21.6 (9-39) | 62.7 | 62.7/40/85.7 | MRS (good 0-2, poor 3-5) | gender, prodrome, presence of psychiatric symptoms; fever in last 3/52; seizures; memory deficits; speech deficits; dyskinesia; decreased GCS; autonomic instability; central hypoventilation; mechanical ventilation; tracheostomy; complication; abnormal MRI; abnormal EEG; abnormal CSF; tumor | older age; long hospital stay; memory deficits at presentation; descreased consciousness; central hypoventilation; complications; abnormal CSF | 12/18  (Poor) |
| Zhang et al 2017 [48] | Case series | 62 | anti-NMDAR | Autoimmune encephalitis |  | 26.3 (10-59) | 53.2 | 46.8/48.6/58.1 (88.4) | MRS (good 0-2, poor 3-6) | Extreme delta brush pattern | None | 11/18  (Poor) |
| **Median** |  | **33** |  |  |  |  |  |  |  |  |  |  |

* = average age could not be determined as mean ages given per antibody subtype

# = no data given regarding age ranges

^ reported more MRIs than there were patients. Not explained in methods why this was the case, but presumably this means multiple MRIs in some patients
